# Supplementary material for: Long-range dispersion-inclusive machine learning potentials for structure search and optimization of hybrid organic–inorganic interfaces
Source: Digit Discov. 2022 Jun 6;1(4):463–75. doi: 10.1039/d2dd00016d (PMC9358753; doi:10.1039/d2dd00016d)
Supplement: DD-001-D2DD00016D-s001 [file DD-001-D2DD00016D-s001.pdf]

Electronic Supplementary Material (ESI) for Digital Discovery.
This journal is © The Royal Society of Chemistry 2022

Supporting Information for:

Long-range dispersion-inclusive machine learning potentials for structure search and optimization of hybrid organic-inorganic interfaces

Julia Westermayr,[1] Shayantan Chaudhuri,[1,2] Andreas Jeindl,[3] Oliver Hofmann,[3] and Reinhard J. Maurer[1]

[1]Department of Chemistry, University of Warwick, CV4 7AL Coventry, UK

[2]Centre for Doctoral Training in Diamond Science and Technology, University of Warwick, CV4 7AL Coventry, UK [3]

Institute of Solid State Physics, Graz University of Technology, 8010 Graz, Austria

E-mail: r.maurer@warwick.ac.uk

Contents

S1 Machine Learning (ML) Models and Datasets **2**

S1.1 Datasets 2

S1.2 Training 3

S1.3 Model Validation 5

S2 ML Optimization **7**

S2.1 Au@C Optimizations 9

S2.2 X2O@Ag Optimization 9

S1 Machine Learning (ML) Models and Datasets

For fitting energies, forces, and Hirshfeld volume ratios, SchNet,[1–3] was used and adapted, which is a continuous-filter convolutional neural network.

S1.1 Datasets

X2O@Ag The training set for X2O@Ag consisted of 8,201 data points taken from Ref.[4] for training. Data points for X2O@Ag were collated from 6,773 single point calculations and 208 geometry optimizations. In addition, we had 6 structure relaxations of the different systems as an additional hold-out test set and further 16 structure relaxations of B2O with systems far away from the surface to test the implementation and accuracy of our method. Geometry optimizations of the hold out test set required about twice as many steps as the geometry optimizations in the training set. The training set was split into 6,800 data points for training, 700 data points for validation, and the rest was used for testing. The model hyper-parameters were sampled on a random grid and optimized according to the performance on the validation set. The final model error was reported on the hold-out test set and is summarized in Table S1.

Au@C As mentioned in the main text, data for Au@C models were obtained from geometry relaxations of Au nanoclusters on a diamond (110) surface. We started with 62 optimizations of cluster sizes of $n = 15$, 20, 30, 35, 40, 45, and 50 which led to a total number of 5,368 data points. Of these data points, we used 4,500 data points for training, 500 for validation, and the rest for testing. In addition, 4 geometry optimizations with a cluster sizes of 20, 30, 40, and 50 were kept as a hold-out test set to test the model performance for optimizations.

For refinement of the training set, we carried out global structure search with initially trained ML models with basin-hopping.[5,6] As starting points for basin-hopping with the initial MLIPs, $ML_{init.}$, we have selected Au nanoclusters (NCs) of different sizes, i.e., the sizes that were featured in the training data set ($n = 15$, 20, 30, 35, 40, 45, 50) and some that were not included ($n = 6$, 25, 28, 44, 66). A basin hopping run was initiated for each NC size. The initial structures of known NC sizes were randomly selected from the optimized structures generated with DFT, i.e., we used data points that made up the training set. Systems of unknown NC sizes were constructed as before with ASE and were placed on the center of the diamond (110) surface.[7] This procedure resulted in 231 structure relaxations. Note that one basin-hopping run comprises several structure relaxations. At the end of each optimization, our algorithm prints the model variance, which was used along with the maximum residual force component to assess the reliability of a structures relaxation. Almost all relaxations with unknown cluster sizes resulted in large model variances, i.e., values $\geq$10 eV, which indicate that the models fail for relaxing these systems. Therefore, data points for adaptive sampling were randomly selected from this set of data points. The relaxations of clusters with sizes known to the MLIPs resulted in smaller model variances and maximum residual forces down to 0.05 eV/Å, hence, those relaxations that resulted in the largest variances were selected to extend the training set. All selected data points were prepared for additional geometry optimizations with DFT. We added each individual step of a geometry optimization to the training

set. In total, 8,893 data points were collected with this procedure. As expected, after the first adaptive sampling run, the variance between the models decreased by a factor of more than 100 for structure optimizations of previously unseen cluster sizes. On average, the model variances is around 0.10 eV with values down to 0.5 meV. The largest model deviation is about 1.0 eV for data points outside the training regime.

MLIPs after the first adaptive sampling run (denoted as $ML_{adapt.1}$) were trained on 7,700 data points for training and 800 data points for validation. The same procedure as before was applied to extend the training set further, but using the $ML_{adapt.1}$ model instead of the $ML_{init.}$ model for initial structure relaxation. In addition, we carried out 243 single point calculations of structures with the largest model errors to let the model know where not to go during optimizations. We collected a total amount of 9,757 data points and final $ML_{adapt.2}$ models were trained on 8,500 data points for training and 800 data points for validation.

S1.2 Training

Energy and Forces Energies and forces were trained with standard SchNet models. The energies and forces that were used for training were obtained after subtraction of van der Waals (vdW) contributions. All reference calculations were carried out with FHI-aims.[8,9]

As already mentioned, two different systems were tested: gold NCs on diamond (110) surfaces (Au@C) and X2O systems on Ag(111) surfaces (X2O@Ag). The energies and forces were trained atom-wise and energies of the whole systems were obtained by summing up atomic contributions. As can be seen from equation 3 in the main text, the resulting energies were mapped to the reference energies. As the systems in the training set were very diverse, total energies varied by a few megaelectronvolts between systems. Thus, energies had to be pre-processed in addition as the current version of SchNet uses data sets saved in an Atomic Simulation Environment (ASE) .db format, which only allows single precision. For X2O@Ag systems we trained energies in the following way:

$$E_{\text{training}} = E_{\text{total,vdW-free}} - \sum_{\text{A}}^{N_A} E_{\text{A}}. \tag{1}$$

N_{A} denotes the number of atoms in a system. The atomic energies that were used for scaling were obtained from reference calculations with the same method that was used to generate the training set, i.e., DFT+vdW^{surf} (see section 2.2.2 in the main text).

Due to the large size of the Au@C systems, the energy deviations between the systems ranged from a few to about 100 MeV. Different ways were tested to train the vdW-free energies and forces. The best model performance was obtained when subtracting the minimum of each cluster size individually. The respective values were saved in the data base and could be added subsequently for predictions. The errors on a hold-out test set for each system for energies and forces can be found in Table S1. After the second adaptive sampling run, a smooth L_1 loss function was applied for training. This was done as the training set for $ML_{adapt.2}$ contained data points with comparably larger forces and energies than most of the data points. Using the L_2 loss function for this dataset would mean that these data points would be weighed comparably large during training, hindering a meaningful model training. Therefore, whenever the model error on a given data point exceeded the mean of

the model error on a given batch size, we switched to the L_1 loss function. The total loss function for energies and forces for $\mathrm{ML}_{\mathrm{adapt.2}}$ thus reads for a given batch size:

$$L^{\mathrm{batch}} = \begin{cases} L_2 \text{ if } \max\left(\left|E_{\mathrm{local}}^{\mathrm{QC}} - E_{\mathrm{local}}^{\mathrm{ML}}\right|\right) < 3 \times \mathrm{mean}\left(\left|E_{\mathrm{local}}^{\mathrm{QC}} - E_{\mathrm{local}}^{\mathrm{ML}}\right|\right) \\ L_1 \text{ if } \max\left(\left|E_{\mathrm{local}}^{\mathrm{QC}} - E_{\mathrm{local}}^{\mathrm{ML}}\right|\right) \geq 3 \times \mathrm{mean}\left(\left|E_{\mathrm{local}}^{\mathrm{QC}} - E_{\mathrm{local}}^{\mathrm{ML}}\right|\right) \end{cases} \tag{2}$$

with

$$L_2 = t_E \left|\left|E_{\mathrm{local}}^{\mathrm{QC}} - E_{\mathrm{local}}^{\mathrm{ML}}\right|\right|^2 + t_F \left|\left|F_{\mathrm{local}}^{\mathrm{QC}} - \frac{\partial E_{\mathrm{local}}^{\mathrm{ML}}}{\partial \mathbf{R}}\right|\right|^2 \tag{3}$$

and

$$L_1 = t_E \left|E_{\mathrm{local}}^{\mathrm{QC}} - E_{\mathrm{local}}^{\mathrm{ML}}\right| + t_F \left|F_{\mathrm{local}}^{\mathrm{QC}} - \frac{\partial E_{\mathrm{local}}^{\mathrm{ML}}}{\partial \mathbf{R}}\right|. \tag{4}$$

$E_{\mathrm{local}}^{\mathrm{QC}}$ and $E_{\mathrm{local}}^{\mathrm{ML}}$ denotes a vector of all energies within a given batch size. Different thresholds between 1-10 were tried for switching between L_1 and L_2 with no significant differences in training performances, hence the original choice of 3 was retained.

Note that the Au@C models obtained after adaptive sampling runs 2 and 3 include geometries that are unlikely to be visited, but are included in the training to let the model know where not to go. Thus, the MAE and RMSE are expected to increase, which does not imply that the performance of the models for geometry optimizations and global structure searches deteriorates. In fact, if we remove 8 outliers from the computation of the MAE and RMSE, the MAE and RMSE for the energy of the "Au@C adaptive2" and "Au@C adaptive3" models decreases by about a third (MAE) and a tenth (RMSE), respectively, and the MAE and RMSE of forces up to half (MAE) and a third (RMSE), respectively, making the errors comparable to previous adaptive sampling runs.

Hirshfeld Volume Ratios The Hirshfeld volume ratios were obtained by dividing the effective atom-in-molecule volumes with the free atomic volumes as given in the main text in equations (1) and (2). Hirshfeld volume ratios were trained atom-wise in a single SchNet model. The SchNet output layer was adapted to fit Hirshfeld volume ratios per atom in one neural network, i.e., in a multi-state neural network, by removing the last pooling layer. The last pooling layer usually sums or averages over the atomic contributions, which is not needed in this case. Hence, multiple, atom-wise values entered the loss function and were mapped directly to the Hirshfeld volume ratios instead of the sum or average of these values. The errors on a hold-out test set for each system are reported in Table S1.

Model Parameters: X2O@Ag For learning energies and forces, a cutoff of 6 Å was used to represent the atoms in their chemical and structural environments. Larger cutoffs were tested, but did not lead to better results, which was expected as long-range interactions were excluded from the training data. We used default parameters in most cases, hence we only state the model parameters that differed from the default: 128 features, 4 SchNet interaction layers to learn the representation, a learning rate of 3×10^{-4}, and a batch-size of 8 was

used. In total, we trained 4 similar models on energies and forces that differed in the trade-off, used to weight energies (t) and forces ($1-t$) during training. Energies were weighted with factors 0.01, 0.03, 0.03, and 0.05 for the different models and the respective force weights were 0.99, 0.97, 0.97, and 0.95.

For learning Hirshfeld volume ratios, a cutoff of 8 Å, a batch size of 2, and a learning rate of 2×10^{-4} was used.

Model Parameters: Au@C For training energies and forces, we used a batch size of 4, 4 interaction layers and 128 features to learn the SchNet representation. A learning rate of $2 \cdot 10^{-4}$ was used and the weights for the energies were set to 0.03, 0.04, 0.04, and 0.05 with weights for forces set to 0.97, 0.96, 0.96, and 0.95, respectively. Besides, we used default parameters of SchNet.

For training Hirshfeld volume ratios, a cutoff of 6 Å, a batch size of 4, a learning rate of $5 \cdot 10^{-4}$, 4 interaction layers to fit the SchNet representation, 128 features, and 25 Gaussian functions for the input layer were used. The rest of the parameters were set to the default values of SchNet.

S1.3 Model Validation

The accuracy of the models for X2O@Ag and Au@C are given in Table S1. In total, 4 energy and force models and one Hirshfeld model were trained for each data set. The errors are reported on a hold-out test set.

System X2O@Ag	Energy [eV] MAE (RMSE)	Forces [eV/Å] MAE (RMSE)	H_A MAE (RMSE)
Model 1	0.017 (0.025)	0.021 (0.035)	
Model 2	0.018 (0.026)	0.025 (0.041)	
Model 3	0.021 (0.030)	0.025 (0.041)	
Model 4	0.021 (0.030)	0.024 (0.041)	
H_A Model			$1.4{\cdot}10^{-4}$ $(7.3{\cdot}10^{-4})$
**Au@C Initial**			
Model 1	0.013 (0.030)	0.021 (0.046)	
Model 2	0.011 (0.029)	0.020 (0.062)	
Model 3	0.011 (0.026)	0.015 (0.037)	
Model 4	0.013 (0.031)	0.019 (0.050)	
H_A Model			$8.1{\cdot}10^{-5}$ $(1.7{\cdot}10^{-4})$
**Au@C adaptive1**			
Model 1	0.020 (0.070)	0.014 (0.032)	
Model 2	0.023 (0.090)	0.014 (0.033)	
Model 3	0.037 (0.055)	0.021 (0.042)	
Model 4	0.028 (0.058)	0.021 (0.041)	
H_A Model			$3.9 \cdot 10^{-5}$ $(1.3 \cdot 10^{-4})$
**Au@C adaptive2**			
Model 1	0.091 (0.561)	0.031 (0.116)	
Model 2	0.138 (0.842)	0.054 (0.260)	
Model 3	0.175 (0.998)	0.062 (0.155)	
Model 4	0.138 (0.869)	0.056 (0.254)	
H_A Model			$6.2{\cdot}10^{-5}$ $(1.7 \cdot 10^{-4})$
**Au@C adaptive3**			
Model 1	0.092 (0.55)	0.040 (0.16)	
Model 2	0.094 (0.55)	0.050 (0.13)	
Model 3	0.12 (1.1)	0.035 (0.26)	
Model 4	0.12 (0.59)	0.066 (0.21)	
H_A Model			$1.1{\cdot}10^{-4}$ $(2.2 \cdot 10^{-4})$

Table S1 Mean absolute errors (MAEs) and root mean-squared errors (RMSEs) of energies, forces, and Hirshfeld-volume ratios on a hold-out test set for X2O@Ag and Au@C.

S2 ML Optimization

The ML models were used for pre-relaxations in case of X2O@Ag and adaptive sampling was carried out for Au@C with initially trained ML models. Thus, as mentioned in the main text briefly, the usually applied *fmax* value of 0.05 eV/Å could not be reached reliably in all structure relaxations, especially when global structure search was used for adaptive sampling with initial ML models.

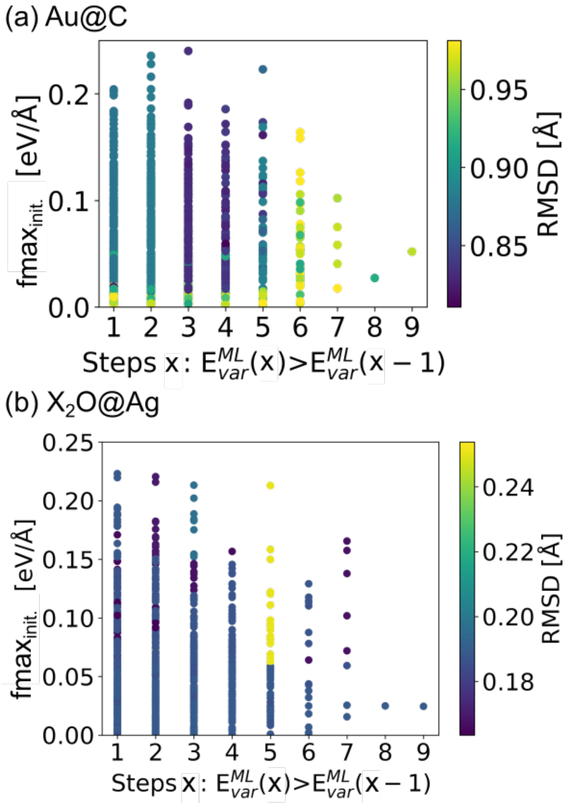

Figure S1 Random grid search of different parameters to stop the structure relaxations with ML models. An initial *fmax*, $fmax_{\text{init.}}$, and the number of consecutive steps, x, after which the variance in energies predicted by the different ML models, $E_{\text{var}}^{\text{ML}}(q)$, was rising, was considered. The color bar shows the root mean squared deviation (RMSD) in Å of the final ML-optimized structure with respect to the DFT-optimized structure.

To this aim we sought to adapt the stopping criteria for structure relaxations to account for the model accuracy. We explored a random grid of 1,000 different stopping criteria using additional structure relaxations of NCs of different sizes for Au@C and the test set of X2O@Ag. We introduced an initial $fmax_{init.}$ in addition to the final *fmax* of 0.05 eV/Å. Further, we took the number of consecutive steps, x, after which the variance in energies, $E_{\text{var}}^{\text{ML}}(q)$, predicted by the query-by-committee models was rising into account. The random grid search is visualized in Fig. S1 (a) and (b) for Au@C and X2O@Ag, respectively.

As can be seen from Fig. S1, in both cases an initial *fmax* in the range of 0.1-0.2 eV/Å in combination with a preliminary termination of the algorithm after three consecutive steps that showed rising energy variances led to the most stable setup and consequently, to structures that were closest to the DFT minimum (lowest root mean squared deviation (RMSD)). We found that the exact value of the initial *fmax* was not critical,

but that it was important to stop the algorithm either after consecutive rising in energy variance or when a final *fmax* of 0.05 eV/Å was reached. Independent of the initial $fmax_{\text{init.}}$, we included another stopping criterion, which terminated the algorithm whenever the model variance exceeded a value of 1 eV or when the $fmax$ jumped to a value that was larger than 2 eV/Å. Both events were observed when model predictions ran into extrapolative regimes and were not reliable anymore. Note that the model variance rises substantially in extrapolative regions, hence, the threshold of 1 eV is not critical, but a value of, e.g., 0.5 eV or 10 eV would lead to identical results or in the worst case one optimization step fewer or more, respectively.

Computational cost ML in combination with external vdW corrections is much more computationally efficient than DFT with vdW corrections. vdW(TS) and vdW^{surf} energy and force evaluations are about 20 times faster than MBD evaluations. MBD further requires much more memory, which represents a computational bottleneck compared to the evaluation of the SchNet-based MLIP.

S2.1 Au@C Optimizations

The structures of the 9 systems with cluster sizes $n = 6$, 15, and 40 are shown in Fig. S2. The number in brackets indicates the energy ranking, i.e., 1 refers to the energetically most favourable structure, while 2 refers to the middle structure and 3 to the energetically least favourable structure.

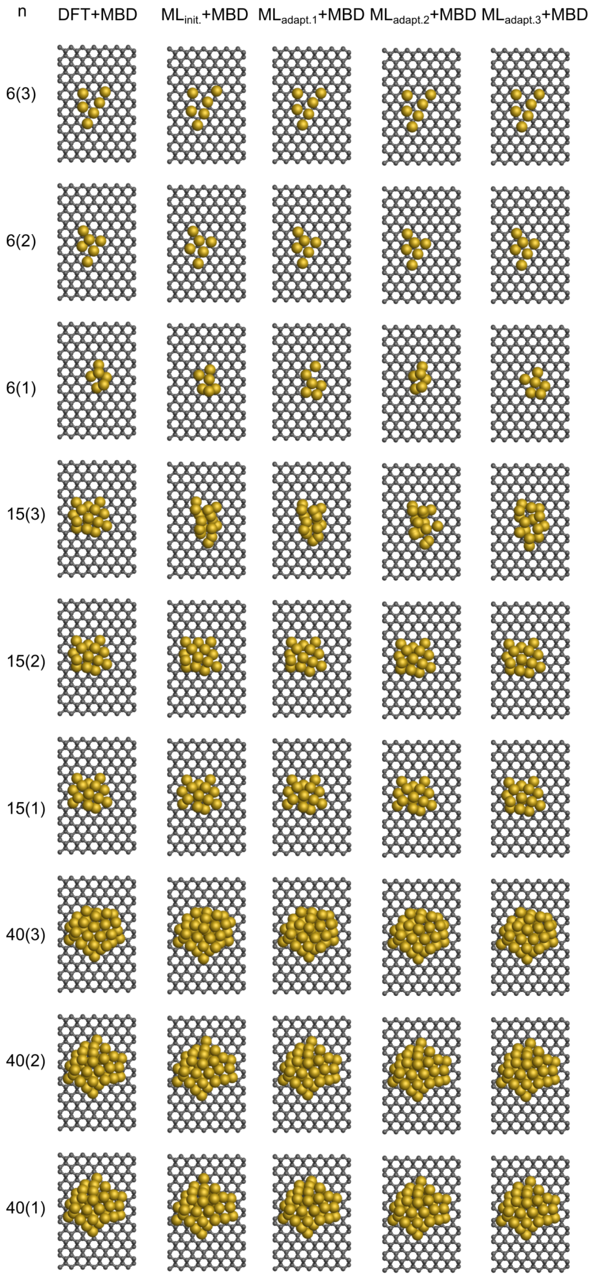

Figure S2 Structures according to Fig. 4d shown from the top view using DFT+MBD, ML$_{init.}$+MBD,ML$_{adapt.1}$+MBD, and ML$_{adapt.2}$+MBD. The number in brackets indicates the energy ranking, i.e., 1 refers to the energetically most favourable structure, while 2 refers to the middle structure and 3 to the energetically least favourable structure.

S2.2 X2O@Ag Optimization

The ML-optimized structures of the test set according to Fig. 5 in the main text are assessed in Fig. S3.

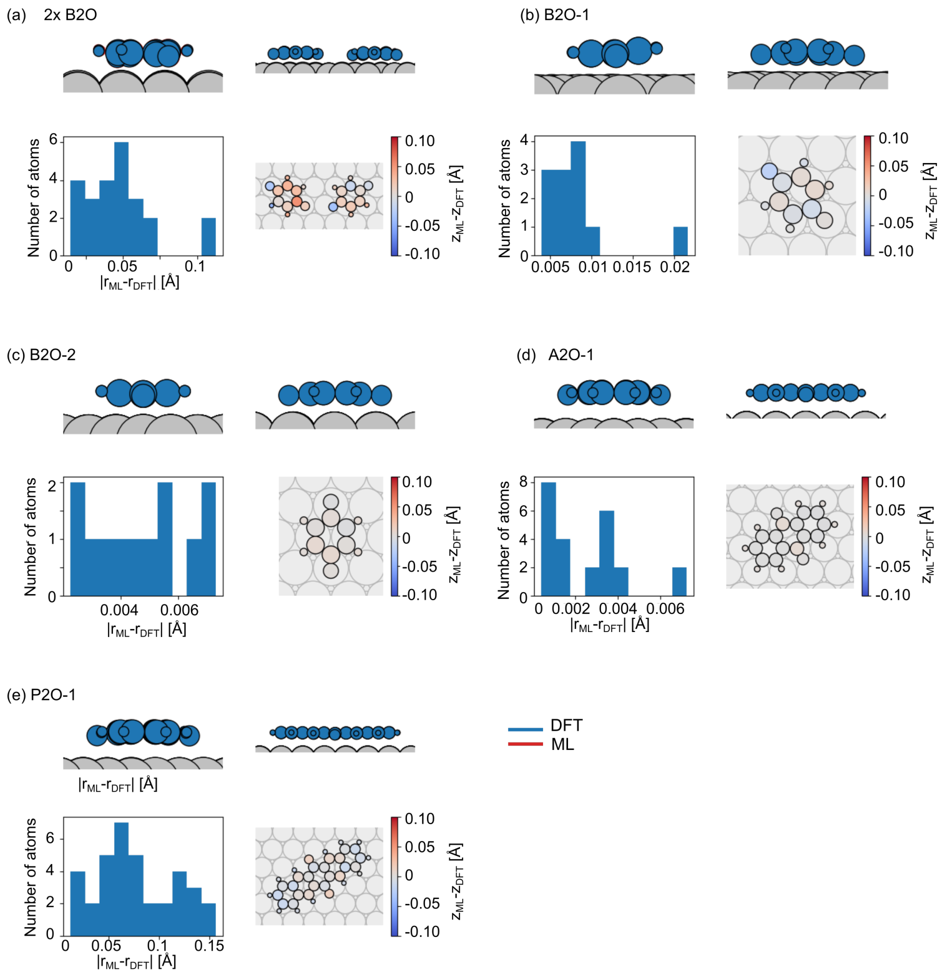

Figure S3 ML+vdW^{surf} structures compared to DFT+vdW^{surf} structure for (a) 2x B2O, (b) B2O-1, (c) B2O-2, (d) A2O-1, and (e) P2O-1 according to Fig. 5 in the main text.

The errors in bond distances and bond angles of the test set structures are shown in Fig. S4.

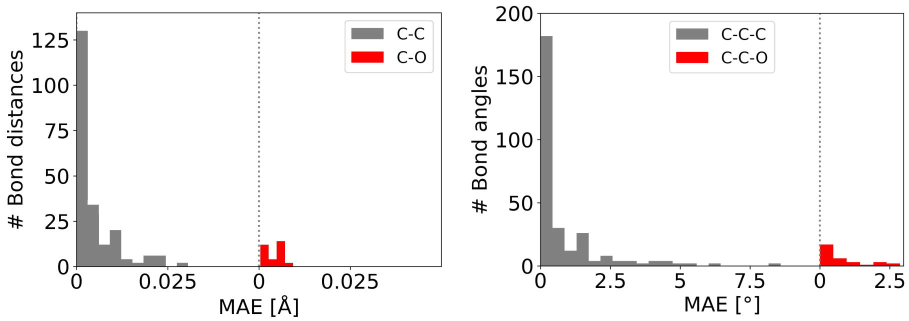

Figure S4 Mean absolute error (MAE) in bond distances (left plot) and bond angles (right plot) of X2O@Ag structures of the test set.

Notes and references

[1] K. T. Schütt, H. E. Sauceda, P. J. Kindermans, A. Tkatchenko and K. R. Müller, *J. Chem. Phys.*, 2018, **148**, 241722.

[2] K. T. Schütt, P. J. Kindermans, H. E. Sauceda, S. Chmiela, A. Tkatchenko and K. R. Müller, Advances in Neural Information Processing Systems, 2017, pp. 992–1002.

[3] K. T. Schütt, P. Kessel, M. Gastegger, K. A. Nicoli, A. Tkatchenko and K.-R. Müller, *J. Chem. Theory Comput.*, 2019, **15**, 448–455.

[4] A. Jeindl, J. Domke, L. Hörmann, F. Sojka, R. Forker, T. Fritz and O. T. Hofmann, *ACS Nano*, 2021, **15**, 6723–6734.

[5] D. J. Wales and J. P. K. Doye, *J. Phys. Chem. A*, 1997, **101**, 5111–5116.

[6] D. J. Wales and H. A. Scheraga, *Science*, 1999, **285**, 1368–1372.

[7] A. H. Larsen, *J. Phys.: Condens. Matter*, 2017, **29**, 273002.

[8] V. Blum, R. Gehrke, F. Hanke, P. Havu, V. Havu, X. Ren, K. Reuter and M. Scheffler, *Comp. Phys. Commun.*, 2009, **180**, 2175–2196.

[9] I. Y. Zhang, X. Ren, P. Rinke, V. Blum and M. Scheffler, *New J. Phys.*, 2013, **15**, 123033.
